# Cultural Adaptation of a Survey to Assess Medical Providers’ Knowledge of and Attitudes towards HIV/AIDS in Albania

**DOI:** 10.1371/journal.pone.0059816

**Published:** 2013-03-27

**Authors:** Shane D. Morrison, Vania Rashidi, Vilson H. Banushi, Namrata J. Barbhaiya, Valbona H. Gashi, Clea Sarnquist, Yvonne Maldonado, Arjan Harxhi

**Affiliations:** 1 Department of Pediatrics, Stanford University School of Medicine, Stanford, California, United States of America; 2 Stanford University School of Medicine, Stanford, California, United States of America; 3 Department of Infectious Diseases, University of Tirana Faculty of Medicine, Tirana, Albania; Vanderbilt University, United States of America

## Abstract

Though the HIV/AIDS epidemic in Southeastern Europe is one of low reported prevalence, numerous studies have described the pervasiveness of medical providers’ lack of knowledge of HIV/AIDS in the Balkans. This study sought to culturally adapt an instrument to assess medical providers’ knowledge of and attitudes towards HIV/AIDS in Albania. Cultural adaptation was completed through development of a survey from previously validated instruments, translation of the survey into Albanian, blinded back translation, expert committee review of the draft instrument, focus group pre-testing with community- and University Hospital Center of Tirana-based physicians and nurses, and test-retest reliability testing. Blinded back translation of the instrument supported the initial translation with slight changes to the idiomatic and conceptual equivalences. Focus group pre-testing generally supported the instrument, yet some experiential and idiomatic changes were implemented. Based on unweighted kappa and/or prevalence adjusted bias adjusted kappa (PABAK), 20 of the 43 questions were deemed statistically significant at kappa and/or PABAK ≥0.5, while 12 others did not cross zero on the 95% confidence interval for kappa, indicating their probable significance. Subsequently, an instrument to assess medical providers’ knowledge of and attitudes toward HIV/AIDS for an Albanian population was developed which can be expanded within Albania and potentially to other countries within the Balkans, which have an Albanian-speaking population.

## Introduction

Albania is a country in Southeastern Europe that has thus far managed to circumvent the growing HIV/AIDS epidemic in Eastern Europe [1]–[9]. The number of Ministry of Health (MoH) reported cases of HIV in Albania remains under 400 in a country of 3.1 million [10]–[13]. However, recent studies suggest that the local epidemic may have a prevalence of up to 150-fold the current estimates while risky behavioral practices are pervasive amongst many of the at-risk groups and the estimated emigration is 27% [10], [12], [14]–[19]. Moreover, current data indicate that barriers to attaining HIV-medical care in Albania are high, especially related to stigma, and one of the most frequent patient-reported barriers was medical providers’ lack of knowledge of HIV/AIDS [20].

Introduction of antiretroviral therapy (ART) and preventative interventions have proved effective around the globe, yet their proper functioning relies on the ability of the healthcare and public health sectors to implement them. Medical professionals’ knowledge of and attitudes towards HIV/AIDS have a substantial impact on the usefulness of these interventions. Low levels of knowledge about HIV/AIDS (including signs, symptoms, transmissibility, etc.) and discriminatory behavior against people living with HIV/AIDS stymies the goals of prevention and treatment regimens; such an atmosphere fosters the likelihood of increased transmission of HIV and decreased adherence to ART [21]–[28]. Understanding medical professionals’ knowledge of and attitudes towards HIV/AIDS is vital to the development of an effective approach to HIV/AIDS within a country. A number of studies have looked at physician knowledge of and attitudes towards HIV/AIDS [25], [27], [29]–[35]. Indeed, recent studies in Central and Eastern Europe, especially in the Balkans, have demonstrated that amongst healthcare workers knowledge of HIV/AIDS is low and discrimination is high [31], [32], [34], [35].

Valid and reliable instruments on medical professionals’ knowledge of and attitudes towards HIV/AIDS in the developing world have been constructed [27], [33]. However, implementation within a new country requires cultural adaptation of the previously validated measure. A number of different studies have proposed methods for cultural adaptation of self-reported measures [36]–[39]. The adaptation consists of a five-step process, 1) Translation of the instrument to the language of the population to be measured, 2) Back translation of the instrument into it original language to check for accuracy, 3) Review of the instrument by a committee of professionals who can critically evaluate the survey, 4) Pre-testing the instrument, and 5) Determination of reliability. Translators that are bilingual in both languages should complete the initial translation. A 2–3-person team should complete the back-translation of the instrument into its initial written language to make sure that none of the information was misinterpreted in the translation. Committee review composed of the team that will implement the measure serves as the final reviewer. The instrument should be checked for semantic, idiomatic, experiential, and conceptual equivalence between the versions. Pre-testing can be approached in a number of ways including a probe technique or focus group pre-testing. Revisions to the instrument should be reviewed carefully before implementation. Lastly, the reliability of the instrument can be measured by having a small group of the study population complete the instrument on multiple occasions and compare the results through statistical approaches [36]–[38].

Based on the rising epidemic of HIV/AIDS in Eastern Europe, the pervasive risky behavioral practices–including low condom use amongst the general population and high rates of needle sharing amongst intravenous drug users–of the predominately younger Albanian population, and preliminary studies in Albania indicating a patient-reported lack of medical professionals’ knowledge of HIV/AIDS, the development of a measure to assess medical professionals’ knowledge of and attitudes towards HIV/AIDS is warranted [10], [12]. Therefore, this study aimed to culturally adapt an instrument that can be used to assess providers’ knowledge of and attitudes towards HIV/AIDS in Albania and potentially expanded to other Albanian speaking populations in the Balkans. Medical professionals with an adequate knowledge of HIV can help prevent of the spread of HIV within and outside of Albania and well as ensure those who are infected receive appropriate care.

## Methods

### Ethics Statement

Approval for the study was granted from the Stanford University Institutional Review Board and the Albanian Ethical Committee. Informed written consent was obtained from each participant prior to enrolling in the study.

### Survey Development

The instrument was developed in conjunction with both the Stanford University School of Medicine and the University Hospital Center of Tirana (UHCT) HIV/AIDS Ambulatory Clinic. The survey addresses the knowledge of and attitudes towards HIV/AIDS. Each of the questions was adapted from previous major studies in the developing world, including the Albanian Behavioral and Biological Surveillance Study Report, the Physician for Human Rights (PHR) Discriminatory Survey, and the Vietnamese Physician Knowledge of HIV/AIDS survey [16], [27], [33]. Each of these measures has been validated in the developing world, but a cultural adaptation was necessitated prior to use in Albania.

The survey has three main portions, 1) demographics, 2) knowledge of HIV/AIDS, and 3) attitudes/discrimination towards HIV/AIDS. The demographics section is brief and inquires about the participant’s age, gender, profession (including years of experience and estimated number of patients with HIV/AIDS under direct care), and religion. This section will mainly be used to inform future analysis. This section of the survey was adapted from the Albanian Behavioral and Biological Surveillance survey and the PHR survey [16], [27]. The questions were kept in the same relative order as they were in previous studies. The next section focuses on knowledge of HIV/AIDS. Participants are prompted with initial basic knowledge questions on HIV presentation, transmissibility, and prevention of HIV/AIDS. This section of the survey was adapted from the Vietnam and PHR studies [27], [33]. The questions were kept in the same relative order as they were in previous studies. The last and largest section of the measure addresses attitudes/discrimination towards HIV/AIDS. This section uses a multi-faceted approach by exploring physician practices, informed consent of patients, physician education on HIV/AIDS, and personal/third-party discrimination against patient with HIV/AIDS. It also elicits perceptions of the HIV prevalence in Albania from medical providers. The PHR survey was the sole informant on this section and the relative order was maintained [27].

**Figure 1:**
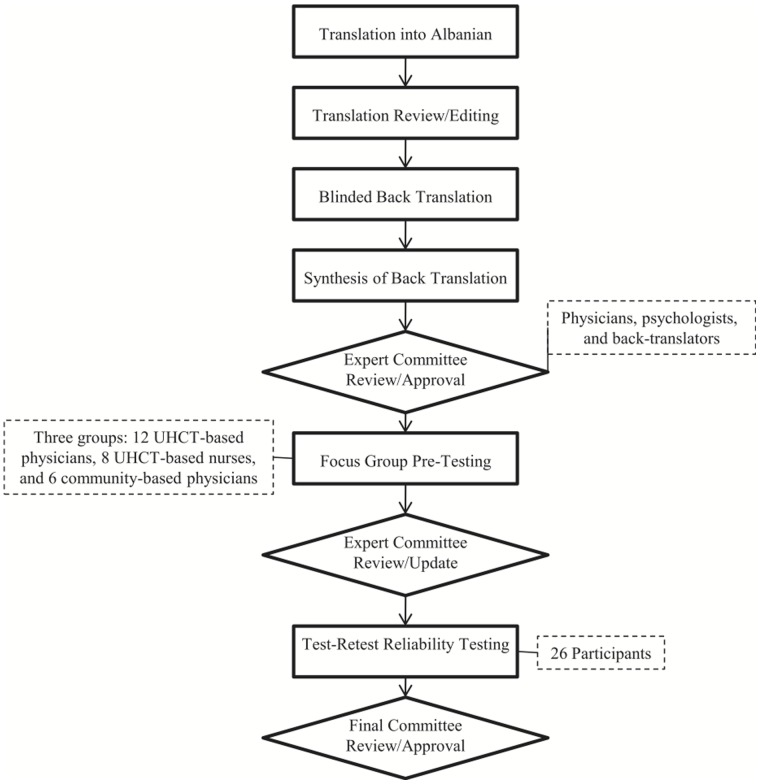
Steps of cultural adaptation of a measure to assess medical providers’ knowledge of and attitudes towards HIV/AIDS in Albania.

A bilingual postdoctoral fellow translated the survey into Albanian. The translator had experience in the medical field, so every attempt to keep the content as close to the original meaning as possible was maintained while incorporating idiomatic changes. Back translation was done by clinic-chosen translators, who verified the accuracy of the translation. The expert committee review (with bilingual Infectious Diseases specialists and psychologists at UHCT) before pre-testing produced the “semi-final” survey. Pre-testing and reliability testing were used to inform the final version of the survey (Figure 1).

### Focus Groups

Focus groups were used for the initial pre-testing of the instrument. Three focus groups were conducted with UHCT-based physicians, UHCT-based nurses, or community-based physicians. The inclusion criteria for the focus groups were: 1) being a current practicing medical provider in Albania with greater than 3 years of experience in their respected field, and 2) have had a current patient population that exceeds 10 patients. Providers were not required to have treated an HIV-positive patient to be in this study. Even without having treated HIV-positive patients, providers can still offer information regarding their knowledge about HIV/AIDS or discrimination of HIV-positive patients that they have witnessed. Providers were excluded if they were not licensed within Albania.

Participants were recruited through departmental or clinic visits. The focus groups consisted of the following participants: 1) UHCT-based physicians – one rheumatologist, two allergy specialists, one anesthesiologist, and eight infectious diseases physicians; 2) UHCT-based nurses – five infectious diseases nurses, one rheumatology nurse, and two intensive care nurses; and 3) community-based physicians – one gastroenterologist, one infectious diseases physician, two family physicians, one neurologist, and one pulmonologist.

The on-site psychologist was trained on the fundamentals of focus group conduction, including the engagement of all participants, the directing of questions back to the topic at-hand, avoidance of leading responses, and flexibility in guiding the process [40].

Written informed consent was obtained prior to the initiation of the focus groups. The survey instrument was then completed by each of the participants. Focus groups explored initial perception of the survey, including material, wording, and length of the instrument, followed by an examination of individual questions. The focus groups were conducted until no new information was brought forth. The focus group were conducted in Albanian and lasted from 1.5–2 hours. The groups were audiotaped for review at a later date.

The on-site psychologist and an on-site physician at the UHCT reviewed audiotapes and participant comments. Transcripts of the participant comments were made in Albanian and English. Information garnered from audiotape review was used to inform the production of the final instrument.

### Test – Retest Reliability Testing

Twenty-six providers – twelve nurses (2 from community-based clinics), nine UHCT-based physicians, and five community-based physicians – were given the revised survey twice within a one-week period. The responses from each of the participants were compared based on Cohen’s kappa. Participants that were included in the focus group pre-testing were excluded from participation in the test-retest reliability testing.

Sample size calculation for the test-retest reliability testing was determined based on the null hypothesis of kappa = 0.0. With a proportion of positive ratings ranging from 0.1–0.9 and an 80% power to detect statistical significance at kappa = 0.50, 25 participants were needed for the study [41].

### Data Analysis

Data was entered electronically and cleaned. Twenty-seven percent of the data was reentered to check for errors: three errors were found, yet these errors were not related to the data to be used for reliability testing.

All data was analyzed with SAS 9.1.3. In determining the test-retest reliability of the measure, unweighted Cohen’s kappa was calculated for each question in the knowledge and discrimination sections of the instrument –43 questions in total [42]. The responses to the questions are categorical and thus no weighting was used–categorical responses that were used were “Yes/Agree,” “No/Don’t Agree,” “Don’t Know,” and “No Answer.” The data is presented with 95% confidence intervals and the standards for strength agreement of kappa: ≤0 = poor, 0.01–0.2 = slight, 0.21–0.4 = fair, 0.41–0.6 = moderate, 0.61–0.8 = substantial, and 0.81–1 = almost perfect [43]. Kappa values under 0.50 are not considered statistically significant at *P*≤0.05 [41]. Prevalence adjusted bias adjusted kappa (PABAK) is reported for questions that were influenced by prevalence or bias [44], [45].

## Results

### Survey Development

Blinded back translation of the survey from Albanian to English following its initial translation showed minimal errors in semantic and idiomatic parameters of the instrument. The survey had to be updated regarding some Albanian idiomatic and conceptual parameters, including “healthcare institution,” “institutional regulations,” “informed consent,” and “prescribe.” The bilingual committee of infectious disease physicians and psychologists made these changes before focus groups were undertaken. Experiential equivalence was maintained by keeping the instrument in the relative order as in their original instruments.

### Focus Groups

Three focus groups were conducted with 12 UHCT-based physicians, 8 UHCT-based nurses, and 6 community-based physicians. For the specialist breakdown of each focus group refer to the methods section. After completion of the survey and consent form, and discussion of the instrument in the focus group setting, the vast majority of the participants agreed that the survey was applicable to their constituency.

Some of the focus group participants had concerns with the length of the survey (>100 questions) and font size (9.5 size font used for paper conservation)–indicating possible experiential concerns. Groups generally felt that there were only 4–5 questions that they had trouble understanding. After focus group transcript review, the committee updated these accordingly. The following are quotes that illustrate the main concerns within the focus groups:

*“The survey was long and I was tired by the time I finished.”* – UHCT-based nurse.

*“Most questions were clear, but I had to read the questions more than once sometimes. If we paid close attention to the questions they were understandable.”* – UHCT-based physician.

*“The percentages were difficult for us to give because we do not have the statistical information about what is going on in Albania. Other centers do this.”* – Community-based physician.

However, the majority of the responses expressed genuine interest and support of the instrument. The following are direct quotes supporting the instrument:

*“The survey was not too long, it was sufficient/long enough. It is feasible to complete it in a reasonable amount of time.”* – UHCT-based physician.

*“The questionnaire should be extended to other fields in medicine.”* – UHCT-based physician.

*“It is very clear, I think, and medical students could understand it too.”* – Community-based physician.

*“It was a valuable survey and I think that this survey should be utilized more within the healthcare centers. Maybe more information about HIV would be beneficial for healthcare providers.”* – UHCT-based nurse.

After the completion of the focus groups, some changes were made to the survey. Some of the main updates included the insertion of a heading at the beginning of the survey that stated that the survey is not a test and approximate answers are sufficient to give, and another statement before questions about patient statistics reminding participants that approximate answers are expected. Clarifications related to what is meant by “types of HIV” and “institutional protocols” were updated before reliability testing was begun. Thus, idiomatic and experiential updates were required before proceeding with reliability testing.

### Test-retest Reliability Testing

Forty-three questions were tested in test-retest reliability testing; however, the entire instrument was completed by each participant at both stages of this testing. The questions fell into three categories: twelve questions for knowledge of HIV/AIDS, ten questions for discrimination against patients with HIV/AIDS, and twenty-one questions on the care and treatment of patients with HIV/AIDS. Seven of the twelve questions in the knowledge section were determined to be statistically reliable at kappa or PABAK ≥0.5 (Table 1): five based on PABAK and two based on unweighted kappa. There were also three questions that do not cross zero in the 95% CI in unweighted kappa. Six of the ten questions in the discrimination section were deemed statistically reliable at kappa or PABAK ≥0.5 (Table 1): all six based on PABAK (one was also significant in unweighted kappa). There were also four questions that were close to PABAK ≥0.5. Seven of the twenty-one questions in the care and treatment of patients with HIV were deemed statistically reliable at kappa or PABAK ≥0.5 (Table 1): all seven based on PABAK (one was also significant in unweighted kappa). Five questions also do not cross zero in the 95% CI in unweighted kappa.

**Table 1:** Kappa and PABAK for questions from the test-retest reliability testing.

Question	Unweighted Kappa	95% CI	PABAK	Kappa Agreement
Knowledge of HIV/AIDS				
1. People can protect themselves from infection with HIV by having good nutrition	0.241	−0.099–0.581	0.384	Fair
2. People can protect themselves from infection with HIV by having one uninfected faithful sexual partner	0.527	0.168–0.887	0.615	Substantial
3. People can protect themselves from infection with HIV by not sharinga toilet seat with a person who has HIV	0.462	0.100–0.824	0.429	Moderate
4. People can protect themselves from infection with HIV by using a condom correctly every time they have sex	−0.074	−0.144– −0.004	0.615	Substantial
5. People can protect themselves from infection with HIV by not sharinga meal with a person who had HIV	0.078	−0.206–0.362	0.231	Fair
6. People can protect themselves from infection with HIV by avoidingmosquito bites	0.400	0.163–0.631	0.154	Fair
7. People can protect themselves from infection with HIV by not sharingneedles and syringes that have previously been used	−0.040	−0.095–0.015	0.846	Almost Perfect
8. Can a pregnant woman infected with HIV transmit the virus to herunborn child?	0.529	0.211–0.847	0.539	Moderate
9. Can a woman with HIV transmit the virus to her newborn child through breastfeeding?	0.506	0.024–0.989	0.769	Substantial
10. Are there more than two types of HIV?	0.462	0.147–0.776	0.462	Moderate
11. Is HIV one example of a sexually transmitted disease?	1.000	N/A	1.00	Perfect
12. Do sexually transmitted diseases increase the probability of beinginfected with HIV?	−0.000	−0.000– −0.000	0.923	Almost Prefect
Discrimination Against Patients with HIV/AIDS				
13. Have you observed others refusing to care for an HIV/AIDS patient?	0.616	0.221–1.000	0.769	Substantial
14. Have you refused to care for an HIV/AIDS patient?	0.490	0.471–0.509	0.923	Almost Perfect
15. Have you observed others refuse an HIV/AIDS patient admission to a hospital?	0.219	−0.103–0.541	0.462	Moderate
16. Have you refused an HIV/AIDS patient admission to a hospital?	0.220	−0.081–0.521	0.769	Substantial
17. Have you observed others give confidential information to a familymember?	0.302	−0.010–0.614	0.385	Fair
18. Have you given confidential information to a family member?	0.139	−0.720–0.350	0.615	Substantial
19. Have you observed others give confidential information to a non-family member?	0.032	−0.190–0.254	0.462	Moderate
20. Have you given confidential information to a non-family member?	0.316	0.015–0.616	0.846	Almost Prefect
21. Have you observed others verbally mistreat an HIV/AIDS patient?	0.171	−0.136–0.479	0.385	Fair
22. Have you verbally mistreated an HIV/AIDS patient?	0.316	0.015–0.616	0.846	Almost Prefect
Care and Treatment of Patients with HIV/AIDS				
23. A person’s HIV status can be determined by his/her appearance	0.005	−0.189–0.198	0.385	Fair
24. Treating someone with HIV/AIDS is a waste of resources	−0.000	−0.000– −0.000	0.923	Almost Perfect
25. A person with HIV/AIDS cannot be treated effectively in this facility	0.266	0.022–0.510	0.077	Fair
26. Medications to treat opportunistic infections may prolong an HIV positive patient’s life	−0.000	−0.000– −0.000	0.846	Almost Perfect
27. It is OK to test someone for HIV without their knowledge	0.299	0.004–0.595	0.231	Fair
28. Many of those who contract HIV/AIDS behave immorally and deserveto have the disease	−0.040	−0.102–0.022	0.692	Substantial
29. If someone has HIV/AIDS his employer/coworkers should be told evenis she/he does not give permission	0.174	−0.062–0.409	−0.154	Slight
30. A health professional with HIV/AIDS should not be working in any areaof the health profession that requires patient contact	−0.026	−0.318–0.265	−0.154	Poor
31. People with HIV/AIDS should not be employed in the health field	0.050	−0.188–0.289	−0.231	Slight
32. All prospective workers should submit to mandatory HIV/AIDS testing	0.319	0.039–0.599	0.154	Fair
33. All prospective health care workers should submit to mandatoryHIV/AIDS testing	0.283	0.001–0.565	0.154	Fair
34. People with HIV/AIDS should be on a separate ward in a hospitalor clinic	0.364	0.132–0.587	0.231	Fair
35. Staff and health care professionals should be told when a patient has HIV/AIDS so they can protect themselves	0.574	0.252–0.895	0.692	Substantial
36. The charts/beds of HIV/AIDS patients should be marked so that clinic/hospital workers know the patient’s status	0.368	0.063–0.672	0.308	Fair
37. The treatment of opportunistic infections in HIV/AIDS patients wastes precious resources	0.288	−0.195–0.771	0.692	Substantial
38. The quality of life of HIV/AIDS patients can be improved with counseling	0.381	−0.007–0.769	0.692	Substantial
39. I can refuse to treat an HIV/AIDS patient to protect myself and family	0.422	0.086–0.758	0.615	Substantial
40. There are circumstances that are appropriate to test a patient for HIV/AIDS without asking the patient for permission/without telling the patient	0.273	0.059–0.487	0.077	Fair
41. There are circumstances where it is appropriate to reveal a persons HIV status to others without the patients knowledge/permission	0.106	−0.250–0.462	0.077	Slight
42. There are circumstances where it is appropriate NOT to reveal a person’s HIV status to him or her	−0.127	−0.321–0.067	−0.154	Poor
43. Relatives and sexual partners of HIV/AIDS patients should be notified for the patients HIV/AIDS status even without his/her consent	0.157	−0.146–0.460	0.077	Slight

### Survey Availability

The survey instrument that was developed in this study was made available to the UHCT HIV/AIDS Ambulatory Clinic. As this is currently the only clinic providing HIV-specialty care in Albania and there is a strong collaboration with the Institute of Public Health in Albania, it is the best point for further implementation and dissemination. The instrument is made available to anyone interested in receiving a copy by contacting the authors. Future discussions with the MoH are planned for implementation.

## Discussion

Cultural adaptation of instruments to future areas of implementation is vital to assure collection of accurate and meaningful data [36]–[38]. Direct translation of an instrument, without proper cultural adaptation, may not efficiently portray the correct semantic, idiomatic, experiential, and conceptual equivalences across instruments–leaving garnered data unfit for proper interpretation. Proceeding through cultural adaptation steps ensures that the measurement tool is culturally suited for the target population. This study went through the steps of cultural adaptation of an instrument to measure medical professionals’ knowledge of and attitudes towards HIV/AIDS in Albania. The process of cultural adaptation used in this study is seen in Figure 1.

In-depth translation of the instrument allowed incorporation of Albanian idiomatic and semantic changes while maintaining its conceptual and experiential English equivalences. The quality of this initial translation was supported by the blinded back translation, which suggested only slight changes to the idiomatic equivalences in the Albanian version in order to maintain accuracy with the initial instrument. Synthesis of the “semi-final” instrument based on review from the expert committee of bilingual physicians and psychologists incorporated only minimal changes to the idiomatic and semantic equivalences of the instrument–mainly related to technical medical terminology.

Focus group pre-testing of the semi-final instrument was met with support and acceptance of the instrument. The main suggestions voiced by participants were related to the length and formatting of the instrument. These comments implied that there might have been the need to change in the experiential equivalence of the instrument; however, the majority of participants declared that the instrument was the proper length for its assessment (see results section). Based on both of these inputs, it was recommended by the committee that there be no changes to the experiential equivalence of the instrument. Still, some of the participants were confused with the role of the survey, the questions asking for approximations, and some technical terms. To adjust for these issues, the committee, based partially on participant suggestion, made changes to the experiential and idiomatic equivalences. First, a line, in bold and all capitals, was added after the introduction indicating that the survey is not a test and is solely interested in participant’s opinions/approximations. Secondly, a line preceding questions exploring patient prevalence approximations was added to let participant know that approximations are expected as answers. Since some participants felt confused on the approximation/statistical questions (see results section), these lines were added to inform participants of the purpose of the questions. Finally, some Albanian idiomatic changes to “institutional protocols” and “types of HIV” were updated prior to reliability testing of the instrument.

Test-retest reliability testing was undertaken to determine the reliability of the instrument. Participants were given the test twice over a one-week period and the reliabilities of 43 questions were determined. Based on this testing, 20 questions were deemed statistically reliable at kappa and/or PABAK ≥0.5 while 12 questions did not cross zero in the 95% CI for unweighted kappa. In total, 32 of the questions could be deemed reliable for further studies (Table 1). Of the total questions that could be reliable in future studies, ten were from the knowledge section (83% of this section), ten were from the discrimination section (100% of this section), and twelve were from the care and treatment section (57% of this section). The vast majority of questions that were not deemed as being reliable were derived from the care and treatment section; this section explores participant opinions of treatment procedures, integration of HIV-positive patients into society, release of confidential material, and other parameters related to the social stigmas of HIV/AIDS. Since this instrument is designed to evolve as participant perceptions evolve in relation to HIV/AIDS, the non-reliability of parts of this section are acceptable as participant perception may have changed within a week, thus it was decided not to alter this section for future studies.

The main limitations that exist in this study are related to the use of only a single translator of the original instrument, the small sample size for test-retest reliability testing, and the un-reliability of certain answers within the instrument. Sometimes in cultural adaptation, more than one translator commissioned to produce a translation; these translations are then synthesized into a common instrument. However, the use of a single initial translator in this study did not jeopardize the instrument, for the back translation, which suggested only minimal changes, and the pre-testing both supported the accuracy and comprehensibility of the instrument. The smaller sample size for the test-retest reliability testing only allowed us to determine statistical significance at unweighted kappa and/or PABAK ≥0.5. This high kappa value may limit the actual number of questions that are considered statistically significant. However, when assessing questions using those not crossing zero on 95% CIs, we were able to show that most questions are reliable. Still, some questions were unreliable; however, as the instrument is meant to evolve with participant perception changes, the unreliable questions were deemed acceptable for use in the study.

In light of the limitations of this study, a final instrument was produced that is ready to be used in a national (Albania) or regional context (Kosovo and Former Yugoslavian Republic (FYR) of Macedonia). This instrument is poised to assess medical providers’ knowledge of and attitudes towards HIV/AIDS within a culturally Albanian region; though there are cultural differences amongst Albanian populations throughout the Balkans, this instrument, constructed in standard Albanian, provides a comprehensible initial survey for further development outside of Albania proper, if needed. As previous studies have identified low levels of knowledge of HIV/AIDS within medical providers within the Balkans, this instrument can be used to assess the providers within culturally Albanian populations and as a starting point for future related studies in this region.

Gathering and synthesizing information related to HIV prevalence, treatment, medical providers’ education, and policy/public health interventions throughout the world has proved difficult, as evidenced by lack of data in many of the large databases constructed by leading international health organizations [2], [4], [9]. One reason for this is due to lack of cultural adaptation of assessment instruments to the population, leading to bias or inadequate data [37], [38]. By culturally adapting this instrument to the Albanian population, inadequacy of data gathering will be circumvented in future implementation of this instrument, something past studies lacked, making this a more rigorous and novel approach. Moreover, with numerous patients, who were receiving care at UCHT HIV/AIDS Ambulatory Clinic, reporting a barrier to them receiving HIV/AIDS specialty care was due to medical provider lack of knowledge of HIV/AIDS, future implementation of this instrument on a national level may have striking public health and policy responses [20]. This could include the restructuring of the medical education to put more emphasis on HIV/AIDS, especially as the epidemic is still on the rise in Eastern Europe. With greater understanding of HIV/AIDS by medical providers, more patients could effectively reach the care and treatment they need, thus quelling the potential spread of the epidemic. As the Albanian population is continuing to grow along with the current high rates of emigration, a healthcare sector effectively prepared to address HIV/AIDS within Albania could have enormous consequences on reducing the spread of the epidemic; something we hope to assess with this new and validated instrument.
